# Multi-Determinants Analysis of Molecular Alterations for Predicting Clinical Benefit to EGFR-Targeted Monoclonal Antibodies in Colorectal Cancer

**DOI:** 10.1371/journal.pone.0007287

**Published:** 2009-10-02

**Authors:** Andrea Sartore-Bianchi, Federica Di Nicolantonio, Michele Nichelatti, Francesca Molinari, Sara De Dosso, Piercarlo Saletti, Miriam Martini, Tiziana Cipani, Giovanna Marrapese, Luca Mazzucchelli, Simona Lamba, Silvio Veronese, Milo Frattini, Alberto Bardelli, Salvatore Siena

**Affiliations:** 1 The Falck Division of Medical Oncology, Ospedale Niguarda Ca' Granda, Milan, Italy; 2 Laboratory of Molecular Genetics, Division of Genetics and Oncogenomics, Institute for Cancer Research and Treatment (IRCC), University of Torino Medical School, Candiolo, Turin, Italy; 3 Service of Biostatistics, Ospedale Niguarda Ca' Granda, Milan, Italy; 4 Laboratory of Molecular Diagnostic, Istituto Cantonale di Patologia, Locarno, Switzerland; 5 Oncology Institute of Southern Switzerland, Ospedale San Giovanni, Bellinzona, Switzerland; 6 Division of Pathology, Ospedale Niguarda Ca' Granda, Milan, Italy; 7 FIRC Institute of Molecular Oncology, Milan, Italy; Dresden University of Technology, Germany

## Abstract

**Background:**

*KRAS* mutations occur in 35–45% of metastatic colorectal cancers (mCRC) and preclude responsiveness to EGFR-targeted therapy with cetuximab or panitumumab. However, less than 20% patients displaying wild-type *KRAS* tumors achieve objective response. Alterations in other effectors downstream of the EGFR, such as BRAF, and deregulation of the PIK3CA/PTEN pathway have independently been found to give rise to resistance. We present a comprehensive analysis of *KRAS*, *BRAF*, *PIK3CA* mutations, and PTEN expression in mCRC patients treated with cetuximab or panitumumab, with the aim of clarifying the relative contribution of these molecular alterations to resistance.

**Methodology/Principal Findings:**

We retrospectively analyzed objective tumor response, progression-free (PFS) and overall survival (OS) together with the mutational status of *KRAS*, *BRAF*, *PIK3CA* and expression of PTEN in 132 tumors from cetuximab or panitumumab treated mCRC patients. Among the 106 non-responsive patients, 74 (70%) had tumors with at least one molecular alteration in the four markers. The probability of response was 51% (22/43) among patients with no alterations, 4% (2/47) among patients with 1 alteration, and 0% (0/24) for patients with ≥2 alterations (p<0.0001). Accordingly, PFS and OS were increasingly worse for patients with tumors harboring none, 1, or ≥2 molecular alteration(s) (p<0.001).

**Conclusions/Significance:**

When expression of PTEN and mutations of *KRAS*, *BRAF* and *PIK3CA* are concomitantly ascertained, up to 70% of mCRC patients unlikely to respond to anti-EGFR therapies can be identified. We propose to define as ‘*quadruple negative*’, the CRCs lacking alterations in *KRAS*, *BRAF*, PTEN and *PIK3CA*. Comprehensive molecular dissection of the EGFR signaling pathways should be considered to select mCRC patients for cetuximab- or panitumumab-based therapies.

## Introduction

Colorectal cancer (CRC) is the third cause of cancer-related death in the western world [1]. Despite improvements in the therapeutic armamentarium for metastatic CRC (mCRC), the 5-year overall survival (OS) remains poor, with a median survival of 18 to 21 months [2]. Additional drugs, as well as further insights about the mechanisms of resistance, are needed to improve clinical outcome. Treatment options for mCRC nowadays include the chimeric IgG1 monoclonal antibody (moAb) cetuximab and the human IgG2 moAb panitumumab [3], [4]. Both molecules bind to the Epidermal Growth Factor Receptor (EGFR), leading to inhibition of its downstream signaling, providing a meaningful clinical benefit. Objective response rates in unselected populations of mCRC, however, are limited to 8–12% for these agents when used as monotherapy in first [5] and subsequent lines of treatment [4], [6], [7].

We and others have previously shown that somatic *KRAS* mutations (a key effector of the EGFR initiated signaling) can independently impair the efficacy of panitumumab or cetuximab [8]–[10]. This led the U.S. and European health authorities to restrict the use of these agents for patients with wild-type *KRAS* mCRC only [11]–[13]. Although this decision is expected to ameliorate the therapeutic index in this selected population, the objective response rate is still very limited. In fact, it is restricted to 17% (*vs* 0% in *KRAS* mutated) for panitumumab monotherapy [10], to 12.8% (vs 1.2% in *KRAS* mutated) for cetuximab monotherapy [14] and to 59–61% (*vs* 36–33% in *KRAS* mutated) for cetuximab plus either irinotecan- or oxaliplatin-based chemotherapy, respectively [15], [16]. These findings clearly suggest that other factors, such as alterations in other EGFR effectors, including members of the RAS-MAPK or PI3K pathways could drive resistance to anti-EGFR therapy.

BRAF is the principal downstream effector of KRAS [17], [18] and its oncogenic V600E mutation is mutually exclusive with *KRAS* mutations in CRCs [19]. We and others have recently demonstrated that the V600E mutation can also preclude responsiveness to panitumumab or cetuximab in mCRC patients and cellular models of CRC [20]. The *PIK3CA* gene is mutated in approximately 20% of CRCs [21]. *PIK3CA* mutations occurring in the ‘hotspots’ located in exon 9 (E542K, E545K) and exon 20 (H1047R) are oncogenic in CRC cellular models [22], [23]. The *PIK3CA* gene encodes for a lipid kinase that regulates, alongside with KRAS, signaling pathways downstream of the EGFR. PI3K initiated signaling is normally inhibited by PTEN (phosphatase and tensin homologue deleted on chromosome ten). We and others have previously shown that loss of PTEN expression, which occurs in 30% of sporadic cases, is associated with lack of response to cetuximab [24], and that *PIK3CA/PTEN* deregulation may be a biomarker of resistance in *KRAS* wild-type patients [25], [26] and cellular models of CRC [27].

Taken together, data from retrospective analyses show that *BRAF* and *PIK3CA*/PTEN alterations could represent additional tools for selecting mCRC patients for EGFR-targeted treatment [20], [24]–[26]. Nevertheless, a clear identification of which biomarkers should be employed together with *KRAS* in the clinical setting remains to be defined. This is because in these retrospective analyses: a) different determinants were evaluated in each study; and b) an overlapping among some of these biomarkers may occur in individual patients. In the present study, we performed the first comprehensive mutational analysis of *KRAS, BRAF*, and *PIK3CA*, alongside with the evaluation of PTEN expression in a cohort of 132 mCRC treated patients.

## Results

### Distribution and overlap of molecular alterations in individual tumors

**Figure 1:**
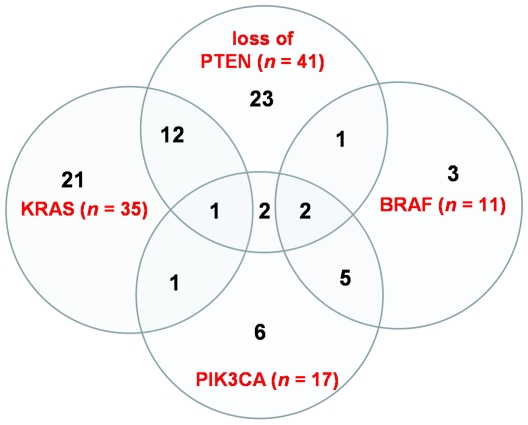
Representation of the distribution of molecular alterations in individual tumors of the 132 patients: mutations of *KRAS* and *BRAF* occurred in a mutually exclusive manner, while an overlapping pattern was observed between other alterations.

As shown in **Figure 1**, analysis of tumors from a cohort of 132 patients (for clinico-pathological features, see **Table 1**) led to the identification of 104 molecular alterations. Specifically, we detected 35 *KRAS* mutations (26.5%), 11 *BRAF* mutations (8.3%), 17 *PIK3CA* mutations (12.3%), and 41 (out of 114 evaluable) loss of PTEN expression (36.0%). Mutations of *KRAS* occurred in codon 12 in 24 cases (68.6%) and in codon 13 in 10 cases (28.6%); a double point mutation involving both codons was detected in one case (2.9%). *PIK3CA* mutations were found either in exon 9 (4 cases) or in exon 20 (13 cases). The 11 mutations of *BRAF* were all V600E substitutions.

**Table 1:** Patients' baseline characteristics.

Number of patients	132
Median age (years) [range]	63.5 [26–85]
Gender (male/female)	86/46
***Primary tumour site***	
Colon	78
Sigma-rectum junction	19
Rectum	31
Other*	4
***EGFR targeted therapy***	
Cetuximab	109
Panitumumab	23
***Previous chemotherapy (%)***	
Irinotecan based	117 (88.6%)
Fluoropyrimidine/capecitabine based	115 (87.1%)
Oxaliplatin based	105 (79.5%)
***No. of previous cancer treatments for advanced disease prior anti-EGFR moAbs (%)***	
None	13 (9.8%)
One	19 (14.4%)
Two	65 (49.2%)
Three	29 (22.0%)
More than three	6 (4.5%)
***Cutaneous toxicity (%)***	
0	21 (15.9%)
1	67 (50.7%)
2–3	37 (28.0%)
Unknown	7 (5.3%)

* Other: in two cases, primary tumor site was small bowel, in one case duodenum and in one case primary tumor sites were multiple (colon and rectum).

Mutations of *KRAS* and *BRAF* occurred in a mutually exclusive manner, while an overlapping pattern was observed among other alterations. The most frequent overlapping fingerprints were PTEN loss and *KRAS* mutations (co-occurring in 13 patients), and *BRAF* and *PIK3CA* mutations (in 7 patients) (**Figure 1**).

### Association of clinical variables and objective tumor response

Among clinical variables (see **Table 1**), only cutaneous toxicity was associated with objective response (Wald's test: p = 0.002; direction of response = 0-1-≥2). Other clinical variables including gender, site of primary tumor (colon, sigmoid-rectum junction, rectum, other sites), and age were not significantly associated with objective tumor response (p = 0.491, 0.490 and 0.904, respectively; p values were obtained by Fisher's exact test for site and gender and by Wald's test for age). Since patients selected in this cohort were treated with mixed lines of previous chemotherapy regimens (although the vast majority received 2–3 previous lines of treatment, see **Table 1**), we also studied the association between the number of previous chemotherapy lines and objective tumor response, showing that this variable did not exert any effect (Wald's test: p = 0.536).

### Multivariate analyses of molecular alterations and objective response

Multivariate analysis including all four molecular alterations (adjusted by cutaneous toxicity and number of previous chemotherapy lines) showed that only *KRAS* mutations and loss of PTEN expression were independently associated with lack of objective response (p = 0.001 and <0.001, respectively) (**Table 2**). Importantly, the Bayesian informative criterion according to Schwarz suggested that a model including *KRAS* mutations and loss of PTEN expression was overall the best strategy in identifying non-responsive patients in our cohort.

**Table 2:** Multivariate analysis of objective response done with exact logistic regression in the cohort of 132 patients evaluated in the study.

Molecular alteration	Odds Ratio of response	CI 95%	*p value*
*KRAS* Mutant *versus* wild type	0.06	0.001–0.469	**0.001**
*BRAF* Mutant *versus* wild type	0.32	0.000–4.175	0.379
*PIK3CA* Mutant *versus* wild type	0.19	0.000–1.701	0.146
PTEN normal *versus* loss	23.89	3.136–997.754	<**0.001**

Odds ratio values are adjusted by score of cutaneous toxicity and number of previous chemotherapy lines.

### Multivariate analyses of molecular alterations and survival

The multivariate Cox analysis for OS confirmed that the role of *KRAS* mutations and loss of PTEN was significant in conferring worse clinical outcome (HR = 1.72, p = 0.043 and HR = 0.54, p = 0.012, respectively), with also *BRAF* mutations exerting a detrimental borderline effect (HR = 2.31, p = 0.093). As for PFS, only *KRAS* mutations were associated with decreased survival (HR = 1.65, p = 0.033), whereas none of the other molecular alterations was demonstrated to independently affect clinical outcome (**Table 3**).

**Table 3:** Multivariate analysis of survival done with exact logistic regression in the cohort of 132 patients evaluated in the study.

Molecular alteration	*PFS Hazard Ratio (CI 95%)*	*p value*	*OS Hazard Ratio (CI 95%)*	*p value*
*KRAS* (mutant *versus* wild type)	1.65 (1.041–2.601)	**0.033**	1.72 (1.017–2.903)	**0.043**
*BRAF* (mutant *versus* wild type)	1.39 (0.521–3.685)	0.513	2.31 (0.867–6.131)	0.093
*PIK3CA* (mutant *versus* wild type)	1.79 (0.801–4.017)	0.156	1.63 (0.815–3.269)	0.166
PTEN (normal *versus* loss)	0.77 (0.501–1.167)	0.213	0.54 (0.332–0.874)	**0.012**

Hazard ratio values are adjusted by score of cutaneous toxicity and number of previous chemotherapy lines.

PFS: progression-free survival; OS: overall survival.

### Multivariate analyses of molecular alterations and clinical outcome in patients with wild-type *KRAS* mCRC

Our cohort consists of retrospective cases and, for this reason, also *KRAS* mutated patients were included in the analysis. After the decision of health authorities to restrict the use of cetuximab and panitumumab to wild-type *KRAS* mCRC [11]–[13], [28], this subgroup has achieved utmost relevance. Accordingly, we focused our analysis on the effect of *BRAF* and *PIK3CA* mutations and loss of PTEN in the remaining 96 wild-type *KRAS* patients. At multivariate analysis, loss of PTEN confirmed a significant association with lack of response (p<0.001), while *BRAF* and *PIK3CA* were not significant (p = 0.265, 0.075, respectively) (**Table 4**).

**Table 4:** Multivariate analysis of objective response done with exact logistic regression among *KRAS* wild-type patients.

Molecular alteration	Odds Ratio of response	CI 95%	*p value*
*BRAF* mutant *versus* wild type	0.24	0.000–3.093	0.265
*PIK3CA* mutant *versus* wild type	0.14	0.000–1.203	0.075
PTEN normal *versus* loss	30.46	3.831–1436.461	<**0.001**

Odds ratio values are adjusted by score of cutaneous toxicity and number of previous chemotherapy lines.

Survival analyses shown in **Table 5** demonstrated that *BRAF* mutations (HR = 3.75, p = 0.015) and loss of PTEN (HR = 0.43, p = 0.009), but not *PIK3CA* mutations (HR = 1.20, p = 0.672), were significantly associated with decreased OS, whereas none of these alterations was significantly associated with PFS.

**Table 5:** Multivariate analysis of survival done with exact logistic regression among *KRAS* wild-type patients.

Molecular alteration	*PFS Hazard Ratio (CI 95%)*	*p value*	*OS Hazard Ratio (CI 95%)*	*p value*
*BRAF* (mutant *versus* wild type)	2.03 (0.66–6.28)	0.218	3.75 (1.29–10.90)	**0.015**
*PIK3CA* (mutant *versus* wild type)	1.45 (0.51–4.14)	0.492	1.20 (0.52–2.78)	0.672
PTEN (normal *versus* loss)	0.81 (0.47–1.39)	0.439	0.43 (0.22–0.81)	**0.009**

Hazard ratio values are adjusted by score of cutaneous toxicity and number of previous chemotherapy lines.

PFS: progression-free survival; OS: overall survival.

### Effect of the number of molecular alterations on clinical outcome

In light of the occurrence of multiple molecular alterations within the same tumor, we investigated our cohort by separating patients according to the actual number of molecular abnormalities in the same tumor, i.e., none *vs* 1 *vs* ≥2 alterations. Because the molecular status of one marker among PTEN, *PIK3CA* and *BRAF* was undetermined in 18 tumors, we decided to perform this analysis in the remaining 114 patients.

**Figure 2:**
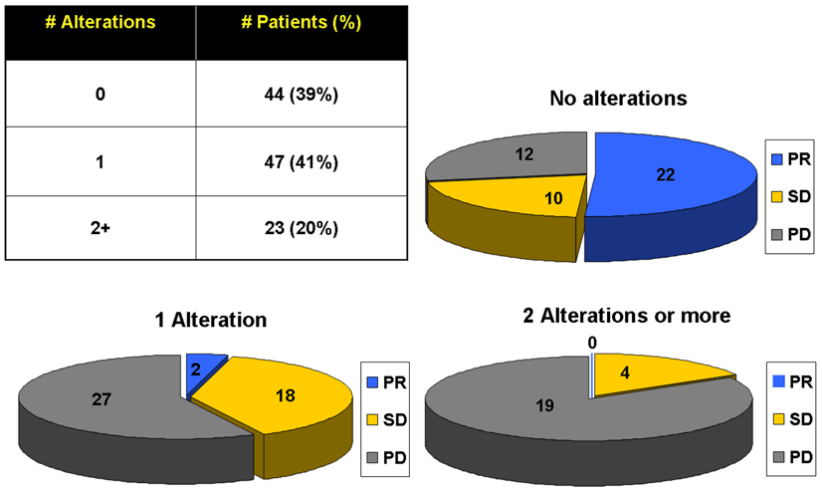
Distribution of the number of mutations (*table*) and response to EGFR-targeted therapy (*pie-charts*) according to the number of molecular abnormalities within individual tumor samples.

**Figure 3:**
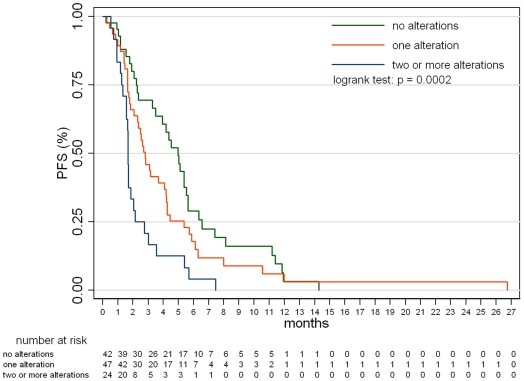
Progression-free survival according to the number of molecular abnormalities within individual tumor samples. Data from the cohort of patients with a known molecular status of all four markers.

The probability of response was 51.1% (22/43) among patients with no alterations, 4.2% (2/47) with 1 alteration, and 0% (0/24) with ≥2 alterations, and these difference were statistically significant (p<0.0001 by Fisher's exact test). **Figure 2** shows distribution of the number of mutations in the cohort and response to EGFR-targeted therapy according to the number of molecular abnormalities within individual tumor samples. The detrimental effect of accumulating alterations was also confirmed by the logistic model, showing that, on average, an unitary increase in the number of alteration(s), would mean a decrease in the odds of response by 96% (odds ratio = 0.04; p<0.00001). Taken together, these findings mean that a necessary but not sufficient condition to reach objective response is to have no more than one of the four molecular alterations. Similarly, survival analyses showed that patients displayed different PFS and OS depending on the number of molecular alterations in their tumors. **Figures 3** **and** **4** show that PFS and OS were increasingly worse for none, 1 or ≥2 molecular alterations (p = 0.0002 for both PFS and OS; logrank test). The pairwise tests adjusted for multiple comparisons documented that a worse clinical outcome was observed for patients with tumors bearing ≥2 alterations *vs* 1 (p = 0.0198 and 0.0213 for PFS and OS, respectively) and ≥2 *vs* none (p = 0.0002 for both PFS and OS), but not for patients with 1 alteration *vs* none (p = 0.3852 and 0.3807 for PFS and OS, respectively). Median PFS was 2.8 months (5.0, 2.8 and 1.7 for patients harboring none, one or ≥2 alterations, respectively); median OS was 9.4 months (14.6, 9.3 and 7.3 for patients harboring none, one or ≥2 alterations, respectively).

**Figure 4:**
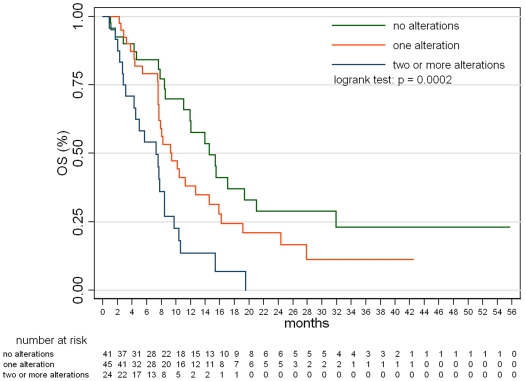
Overall survival according to the number of molecular abnormalities within individual tumor samples. Data from the cohort of patients with a known molecular status of all four markers.

## Discussion

Despite the recent recommendation by ASCO [28] and by health authorities in Europe [11], [12] and US [13] of *KRAS* testing as a diagnostic prerequisite for EGFR-targeted cetuximab- or panitumumab-based therapies for mCRC, the response rate to either of these drugs is limited to less than 20% in wild-type *KRAS* patients [10], [14]. Recent data indicate that *BRAF* or *PIK3CA* mutations may contribute for additional 20–30% of resistance [20], [25], [26]. In addition, also PTEN has been proposed as an independent predictive factor of cetuximab efficacy [24], [25], [27]. However, the relative and overall contribution of each of these molecular alterations to clinical decision making remains unclear. Furthermore, whether and to what extent the occurrence of multiple molecular alterations affects clinical response and patients' survival is presently unknown. *EGFR* copy number assessed by FISH has also been suggested to be predictive of clinical outcome to EGFR-targeted therapies [29]–[33]. However, *EGFR* FISH for mCRC is undergoing inter-laboratory standardization [30] and to avoid the introduction of confounding elements we elected not to carry out this analysis. Here, we exploited the comprehensive molecular analysis of EGFR downstream effectors to ascertain their role in predicting response/resistance to cetuximab or panitumumab in mCRC. By the concomitant assessment of four molecular alterations, we were able to identify up to 70% of non-responder patients, a result that has never been achieved before. Notably, only three patients with tumors carrying a single alteration were in the subgroup of responders, (two patients with *KRAS* mutations and one patient with loss of PTEN expression), whereas no others showed any alteration (“quadruple negative” tumors). This suggests that previously reported outliers, i.e., very uncommon cases of mCRC with *KRAS* mutations responding to therapy [9], [14], [29], [34] may be patients harboring only one of these molecular alterations, thus not concurrently deregulating both MAPK and PI3K pathways.

Our data indicate that single or multiple mutations of *KRAS*, *BRAF*, or *PIK3CA* unfavorably affect clinical outcome to cetuximab- or panitumumab-based therapies; however, the possibility that these molecular alterations could be negative prognostic biomarkers independently from targeted therapies should be taken into account. The RASCAL retrospective study conducted on 2721 CRC patients indicated that the presence of *KRAS* mutations is associated with a 26% increased risk of fatal outcome [35]. However, conflicting data on the same topic have been recently published. In a phase III trial reported by Karapetis et al. [14], clinical benefit in patients with wild-type *KRAS* mCRC was found in cetuximab treated patients but not in control patients treated with best supportive care only, thus indicating that the benefit was not due to a prognostic effect of *KRAS*. Moreover, Roth et al. [Proc Am Soc Clin Oncol Gastrointestinal Cancers Symposium 206, 2009; Abstr 288] tested the prognostic value per stage of *KRAS* and *BRAF* mutations using CRC tumor samples from the adjuvant PETACC3 trial, and they found no significant effects on relapse-free survival for both mutations, neither in stage II nor in stage III. Studies assessing the impact of other molecular alterations rather than *KRAS* mutations are limited. As for the prognostic role of *PIK3CA* and *BRAF*, in a study including 586 patients by Barault et al., decreased rates of 3-year survival were associated with mutations of at least one gene among *KRAS*, *BRAF* and *PIK3CA* [36]. A recent report by Tol et al. found that the presence of the BRAF V600E mutation was a negative prognostic marker in 516 patients with metastatic colorectal cancer treated with capecitabine, oxaliplatin, and bevacizumab based regimens [37]. Finally, Ogino et al. reported that, in a series of 450 patients with stage I–III CRC who underwent curative surgery, tumor *PIK3CA* mutation was associated with shorter cancer-specific survival. Such adverse effect of *PIK3CA* mutation on prognosis was consistent across most strata of clinical and tumoral predictors of patient outcome. Interestingly, this adverse effect was mainly limited to patients with *KRAS* wild-type tumors [38]

**Figure 5:**
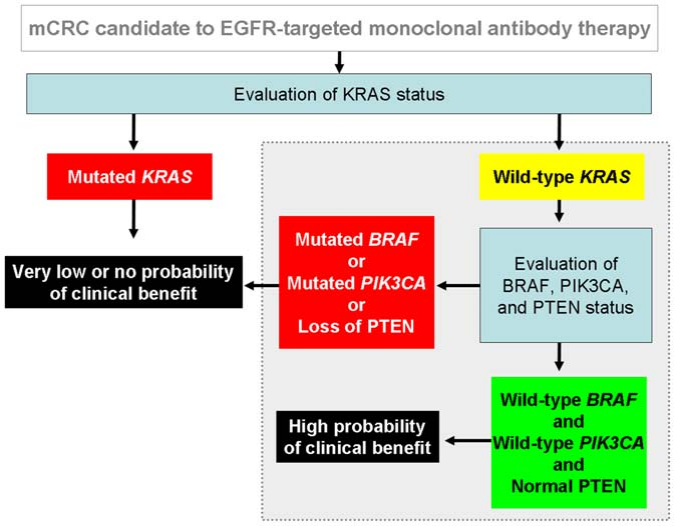
Algorithm of molecular diagnostics based on data discussed in this study for patients with mCRC candidates to cetuximab- or panitumumab-based therapies. The area marked in grey within the dotted line box describes the hypothesis generated in this study.

In conclusion, we document that concomitant detection of *KRAS, BRAF* and *PIK3CA* mutations and evaluation of loss of PTEN expression in mCRC patients has remarkable clinical implications by increasing the ability to predict the outcome to EGFR-targeted therapies. In light of the nature of our patient series, the most reliable indicator of the predictive value of biomarker(s) is objective tumor response. Interpretation of survival analyses should indeed take into account a possible limitation due to patients treated with mixed previous line(s) of chemotherapy including a 10% (13/132) of patients treated with first-line cetuximab monotherapy. On the other hand, the study of such patients represents a unique opportunity to ascertain the predictive value of a given biomarker without the influence of chemotherapy, either concurrent or previous, as well as of selection exerted by other treatments. In light of these considerations, we propose here a new algorithm for deciding the clinical use of EGFR-targeted monoclonal antibodies that is based on objective response rates (**Figures 5** **and** **6**). This novel approach deserves validation in prospective studies with cetuximab- or panitumumab-based therapies in mCRC prior to have an impact as clinical practice-changing. Importantly, we found that approximately 20% of mCRC non-responders do not harbor mutations of *KRAS*, *BRAF*, *PIK3CA* nor loss of PTEN expression and we propose to define these tumors as “quadruple negative”. The lack of response in quadruple negative patients may be due to multiple reasons including but not restricted to: a) the limited sensitivity of current sequencing methods in detecting point mutations in DNA extracted from FFPE tumors [39]; b) the oncogenic deregulation of the same four genes by mechanism other then mutations (such as amplification as reported for *PIK3CA*); c) the occurrence of alterations in other key elements of the EGFR-dependent signal cascade (such as for example AKT or MAPK); and d) the presence of genetic alterations in tyrosine kinase receptors other than EGFR, providing an alternate pathway of survival and/or proliferation. Further molecular dissections of the EGFR-initiated oncogenic signaling cascade are likely to be helpful in improving the tailoring of EGFR targeted therapies. Overall, our results underscore the relevance of using molecular-based algorithms to shift the treatment of solid tumors into the era of personalized cancer medicine.

**Figure 6:**
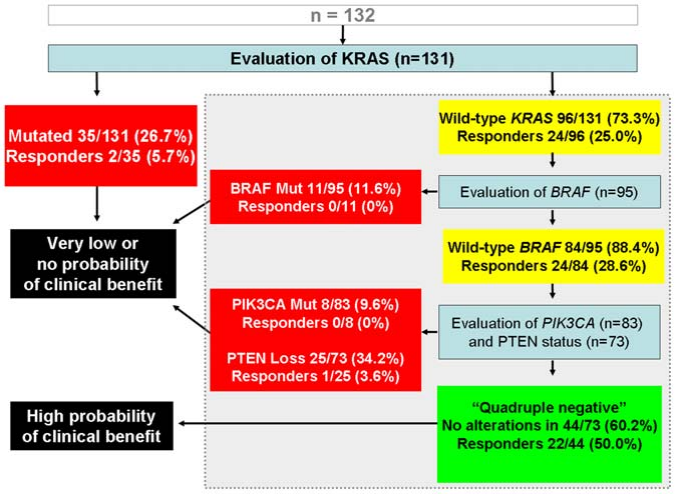
Following evaluation of *KRAS* status in individual tumors, enhancement of predictability of clinical benefit may derive from assessment of the status of *BRAF*, *PIK3CA* and PTEN, as simulated here based on analyses of subgroups from the present cohort (n = 131). We propose to define as “quadruple negative” the mCRCs lacking alterations in *KRAS*, *BRAF*, PTEN and *PIK3CA*.

## Materials and Methods

### Ethics statement

Samples were collected according to the ethical requirements and regulations and obtaining written consent as approved by the review board of the Ospedale Niguarda Ca' Granda, Milano, Italy and of the Ospedale San Giovanni, Bellinzona, Switzerland.

### Patient population and treatment regimens

We retrospectively analyzed 132 patients with EGFR-positive mCRC at Ospedale Niguarda Ca' Granda (Milan, Italy), at the Oncogenomics Center, Institute for Cancer Research and Treatment, (Candiolo, Italy), or at the Institute of Pathology (Locarno, Switzerland). Patients gave informed consent and were treated with panitumumab- or cetuximab-based regimens at Ospedale Niguarda Ca' Granda or at the Oncology Institute of Southern Switzerland (Bellinzona, Switzerland). Patients were selected based on evidence that treatment outcome could be attributable only to administration of panitumumab or cetuximab. Patients' clinical characteristics are reported in **Table 1**. With the exception of 13 patients who received cetuximab as frontline monotherapy [5], the others had failed at least one prior chemotherapy regimen. Twenty-three (17.4%) received panitumumab monotherapy, fifteen (11.4%) patients cetuximab monotherapy, and ninety-four (71.2%) cetuximab plus irinotecan-based chemotherapy. For patients who progressed on irinotecan-based chemotherapy, cetuximab was administered with irinotecan at the same dose and schedule to which they were resistant. Treatment was continued until progressive disease (PD) or toxicity occurred, as per standard criteria [40].

### Clinical evaluation and tumor response criteria

Clinical response was assessed every 6–8 weeks with radiological examination (CT or MR) according to RECIST criteria. Objective tumor responses were classified into partial response (PR), stable disease (SD), and PD. Patients with SD or PD were also defined as non-responders. Response to therapy was also evaluated retrospectively by independent radiologists.

### Molecular analyses

Formalin-fixed paraffin-embedded (FFPE) tumor blocks were reviewed for quality and tumor content. A single representative block, from either the primary tumor or the liver metastasis, depending on availability, containing at least 70% of malignant cells, was selected for each case. Genomic DNA was extracted using the QIAamp Mini kit (Qiagen, Chatsworth, CA, USA) according to the manufacturer's instructions. Molecular analyses were performed on tissue sample from primary tumor used for initial diagnosis in 130 out of 132 cases. In two cases only the analysis was performed on metastatic sites (liver).

### PTEN expression

PTEN protein expression was evaluated by immunohistochemistry on 3 µm FFPE tissue sections as reported [24] with some modifications. Briefly, anti-PTEN Ab4 (Thermo Fisher Scientific, CA, USA) with 1∶200 dilution and PTEN Ab2 (Neomarkers, Fremont, CA) with 1∶50 dilution were used at the Niguarda Hospital and at the Institute of Pathology of Locarno, respectively. PTEN protein expression was mainly detected at cytoplasmic level, while very few cases showed also nuclear positivity. Tumors were considered negative, i.e. with loss of PTEN expression, when absence or reduction of immunostaining was seen in more than 50% of cells as compared with internal controls (i.e. vascular endothelial cells and nerves). Normal endometrium was used as external positive control. The evaluations were performed by two independent pathologists without knowledge of clinical data or results of molecular analyses.

### Mutational analysis of *KRAS*, *BRAF* and *PIK3CA* in tumor samples

We searched for *KRAS* (exon 2), for *BRAF* (exon 15) and for *PIK3CA* (exons 9 and 20) mutations. *KRAS* exon 2 includes codons 12 and 13, *BRAF* exon 15 includes codon 600, *PIK3CA* exon 9 includes codons 542 and 545 and *PIK3CA* exon 20 includes codon 1047, where the large majority of mutations occur in these genes [29]. The list of primers used for mutational analysis is available from the authors upon request. All samples were subjected to automated sequencing by ABI PRISM 3730 (Applied Biosystems, Foster City, CA, USA). All mutated cases were confirmed twice with independent PCR reactions.

### Statistical analyses

All data were analyzed with the suitable descriptive statistical methods, after checking their distributions by means of the Shapiro-Wilk test. Cross-tabulations of qualitative variables were analyzed with the Fisher's exact test, while comparisons between continuous variables were carried out with Student's t or Mann-Whitney U tests. Logistic regression with Wald's test, and exact logistic regression (dealing with one-way causation, such as the case where all patients in a group show a positive or negative outcome), with exact p-value (as the probability of observing a more extreme value with respect to sufficient statistics for a given regression parameter) were used to assess univariate and multivariate analysis with binary endpoint. The survival analysis was performed with the Kaplan-Meier survivor function followed by logrank test, and with the Cox model; proportional hazard assumption was checked using the Schoenfeld residuals. Statistical significance was assumed for p<0.05. All the statistical analyses were done using Stata/SE 10.1 (the StataCorp, College Station,TX-USA).
